# Research and Remedies

**Published:** 1913-01-25

**Authors:** 


### RESEARCH AND REMEDIES.

### Infectious Jaundice.

During the South African War there was at one time quite an epidemic of mild catarrhal jaundice among our troops, and mild epidemics of a similar kind are not very uncommon here in Britain. As a rule, these are attributed to some disturbing element in the food or drink of those who exhibit the symptoms; but Dr. Leonard Guthrie has lately investigated a small epidemic in the Kilburn district, in which he obtained evidence of a more personal contagiousness. At the conclusion of a survey of these cases and of the literature, published in the *British Journal of Children's Diseases*, he states: That mild epidemics of so-called catarrhal jaundice have been especially prevalent in this country during the past three years. That although mild in character hitherto, it is possible that at any time they might become more formidable. (This form of epidemic jaundice is not separable by any hard-and-fast line from Weil's Disease, of which it may quite conceivably be a benign type.) That this catarrhal jaundice must be regarded as due to an acute hepatitis resembling mumps in some particulars. The prognosis in any case of jaundice in childhood, whether sporadic or epidemic, must be guarded, for acute yellow atrophy of the liver begins in just the same way; and, though it is very rare, the possibility should not be lost sight of. Finally, Dr. Guthrie remarks, it is hardly possible to return an emphatic negative to the question "Is jaundice catching?"

### Where to Amputate for Gangrene.

In selecting the exact site of amputation when dealing with a case of gangrene of a limb the surgeon is always at a difficulty, because he wants to amputate as low as possible and yet to avoid the complication of having subsequently to operate again at a higher level. In consequence of this, surgical teaching inclines rather to amputations so far above the seat of visible mortification that the patient is secured against a second operation and against sloughing of the flaps. Thus many amputations are done unnecessarily high. McKinley, in the *International Journal of Surgery*, describes a plan which he has invented for choosing the site of amputations rationally. With no tourniquet applied, he dissects down on to the main vessels well below the upper level of gangrene, and then follows them up until he finds them no longer occluded. A tourniquet is in readiness for application if necessary. Having ascertained the lowest limit of patency of the artery, he then plans his amputation in accordance with the circumstances. It sounds simple and practical, and the author says it works well.

### The Causes of Ectopic Gestation.

It has long been supposed that tubal constrictions or obstructions, generally the results of past inflammation, dispose to the occurrence of tubal pregnancy. Absolute proof of this thesis is hardly to be expected; but a case recently reported by Bandler, of New York, certainly supports it, and is very interesting from two other points of view. A young woman, recently married, consulted him for symptoms suggestive of a possible ectopic gestation. On performing an exploratory laparotomy a hæmorrhagic and enlarged ovary was found, but the tube was apparently healthy. The ovary and tube were removed, and on subsequent examination it was found that the gestation was really in the tube after all, but was so early that it had caused no enlargement. The entire ovum measured 3¾ by 3½ by 2 millimetres, and did not nearly fill the lumen of the tube. Further still, it was shown by serial sections that the ovum was in a blind alley or diverticulum, leading off from the true lumen of the tube. This, of course, accounted for its stoppage and failure to reach the uterus. It seemed probable that in a little while the growth of the ovum would have broken down the inner wall of the diverticulum and so restored the ovum to the true lumen of the tube.

### Medicinal Treatment of Adenoids.

Broadly speaking, operation is the only treatment generally adopted in this country for adenoid vegetations, though breathing exercises, cod-liver oil, iron preparations, and climatic treatment are often advised in mild cases. According to the *Medical Review*, Lapeyre, Roos, and Lucas-Championnière are all agreed that potassium iodide, or iodine itself, taken internally is capable of causing retrogression of these troublesome growths. The doses recommended are two to four grains (for children) twice or three times a day; and the authorities mentioned are satisfied that the benefit of this medication is not by any means confined to those in whom there is reason to suspect a syphilitic taint. It is necessary to persevere with the treatment for two or three months at least, and Lucas-Championnière states that even when it does not actually cure, it at least renders operative measures much more satisfactory and successful.

### The Pathology of Acute Iritis.

The orthodox teaching of the day is that the two common causes of acute iritis are rheumatism and gonorrhœa, and that there are a few much rarer causes of this lesion. Dr. Cobbledick expresses in the *Ophthalmoscope* his confident belief that most if not all the cases that have been classified as rheumatic are really gonorrhœal in origin, and that the gonococcus is by far the most common cause of *acute* iridocyclitis. He lays stress on the necessary consequence of this view—namely, that it is the ophthalmic surgeon's duty to urge on all his patients (suffering from acute iridocyclitis) the importance of special treatment for the urethral disease in order to lessen the possibility of recurrence of the eye trouble. In treatment Dr. Cobbledick relies on leeches and aspirin, together with a gonococcus vaccine of mixed strains in doses of twenty millions. Incidentally, he found that the vaccine cut short the joint manifestations in cases which had been supposed to be rheumatic.

